# Novel bacteriocyte-associated pleomorphic symbiont of the grain pest beetle *Rhyzopertha dominica* (Coleoptera: Bostrichidae)

**DOI:** 10.1186/s40851-017-0073-8

**Published:** 2017-08-19

**Authors:** Genta Okude, Ryuichi Koga, Toshinari Hayashi, Yudai Nishide, Xian-Ying Meng, Naruo Nikoh, Akihiro Miyanoshita, Takema Fukatsu

**Affiliations:** 1 National Institute of Advanced Industrial Science and Technology (AIST), Tsukuba, 305-8566 Japan; 2 Department of Biological Sciences, Graduate School of Science, University of Tokyo, Tokyo, 113-0033 Japan; 3 Institute of Agrobiological Sciences Ohwashi, National Agriculture and Food Research Organization (NARO), Tsukuba, 305-8634 Japan; 4 Department of Liberal Arts, The Open University of Japan, Chiba, 261-8586 Japan; 5 Food Research Institute, National Agriculture and Food Research Organization (NARO), Tsukuba, 305-8642 Japan; 6 Graduate School of Life and Environmental Sciences, University of Tsukuba, Tsukuba, 305-8572 Japan

**Keywords:** *Rhyzopertha dominica*, Lesser grain borer, Bacterial symbiont, Bacteroidetes, Bacteriocyte, Pleomorphism, L-form bacteria

## Abstract

**Background:**

The lesser grain borer *Rhyzopertha dominica* (Coleoptera: Bostrichidae) is a stored-product pest beetle. Early histological studies dating back to 1930s have reported that *R. dominica* and other bostrichid species possess a pair of oval symbiotic organs, called the bacteriomes, in which the cytoplasm is densely populated by pleomorphic symbiotic bacteria of peculiar rosette-like shape. However, the microbiological nature of the symbiont has remained elusive.

**Results:**

Here we investigated the bacterial symbiont of *R. dominica* using modern molecular, histological, and microscopic techniques. Whole-mount fluorescence in situ hybridization specifically targeting symbiotic bacteria consistently detected paired bacteriomes, in which the cytoplasm was full of pleomorphic bacterial cells, in the abdomen of adults, pupae and larvae, confirming previous histological descriptions. Molecular phylogenetic analysis identified the symbiont as a member of the Bacteroidetes, in which the symbiont constituted a distinct bacterial lineage allied to a variety of insect-associated endosymbiont clades, including *Uzinura* of diaspidid scales, *Walczuchella* of giant scales, *Brownia* of root mealybugs, *Sulcia* of diverse hemipterans, and *Blattabacterium* of roaches. The symbiont gene exhibited markedly AT-biased nucleotide composition and significantly accelerated molecular evolution, suggesting degenerative evolution of the symbiont genome. The symbiotic bacteria were detected in oocytes and embryos, confirming continuous host–symbiont association and vertical symbiont transmission in the host life cycle.

**Conclusions:**

We demonstrate that the symbiont of *R. dominica* constitutes a novel bacterial lineage in the Bacteroidetes. We propose that reductive evolution of the symbiont genome may be relevant to the amorphous morphology of the bacterial cells via disruption of genes involved in cell wall synthesis and cell division. Genomic and functional aspects of the host-symbiont relationship deserve future studies.

## Background

Many insects harbor symbiotic bacteria in their gut, body cavity, and/or cells [1]. Some bacterial symbionts are indispensable for the growth, survival, and reproduction of their insect hosts through synthesizing essential nutrients [2, 3], assisting food digestion [4, 5], or providing food sources [6, 7]. Other bacterial symbionts are not essential, but nonetheless influence a variety of host biological traits, such as susceptibility to natural enemies [8, 9], tolerance to environmental stresses [10, 11], resistance to noxious chemicals [12, 13], adaptation to specific food plants [14, 15], or sex ratios and related reproductive traits [16, 17]. The most intimate host–symbiont associations often involve development of a specific symbiotic organ, called the bacteriome, consisting of specialized cells for symbiosis, called the bacteriocytes, the cytoplasm of which harbors symbiotic bacteria [1, 18, 19].

Beetles, comprising the largest insect order Coleoptera, are characterized by sclerotized exoskeleton, including thick and hard forewings, known as elytra [20]. Some beetles cause significant damage to stored cereals, beans, seeds, spices, dried fruits, and other durable commodities, and are thus regarded as stored-product pests [21, 22]. Probably relevant to their peculiar ecological niche, namely continuous living on non-fresh and monotonous food sources under low-humidity conditions, many, if not all, stored-product pest beetles are associated with symbiotic microorganisms (reviewed in [1]). Relatively well-studied examples are the grain weevils *Sitophilus oryzae, S. granarius* and allied species (Curculionidae), which harbors the γ-proteobacterial endosymbiont, “*Candidatus* Sodalis pierantonius” in its bacteriome [23–25]; the cigarette beetle *Lasioderma serricorne* and the drugstore beetle *Stegobium paniceum* (Anobiidae) associated with yeast-like symbiotic fungi, *Symbiotaphrina* spp., which are found both endocellularly in intestinal epithelial cells and extracellularly in the intestinal cavity [26–28]; and the flour beetle *Tribolium confusum* (Tenebrionidae) infected with an α -proteobacterial *Wolbachia* endosymbiont that infects a variety of cells and tissues and causes reproductive phenotypes such as cytoplasmic incompatibility [29–31]. Pioneering early research also provided detailed descriptions of well-developed bacteria-containing symbiotic organs in other stored-product pests belonging to such beetle families as the Silvanidae and the Bostrichidae [32–36], but the microbiological aspects of these symbiotic relationships have remained unstudied in the decades since the original descriptions.

**Fig. 1:**
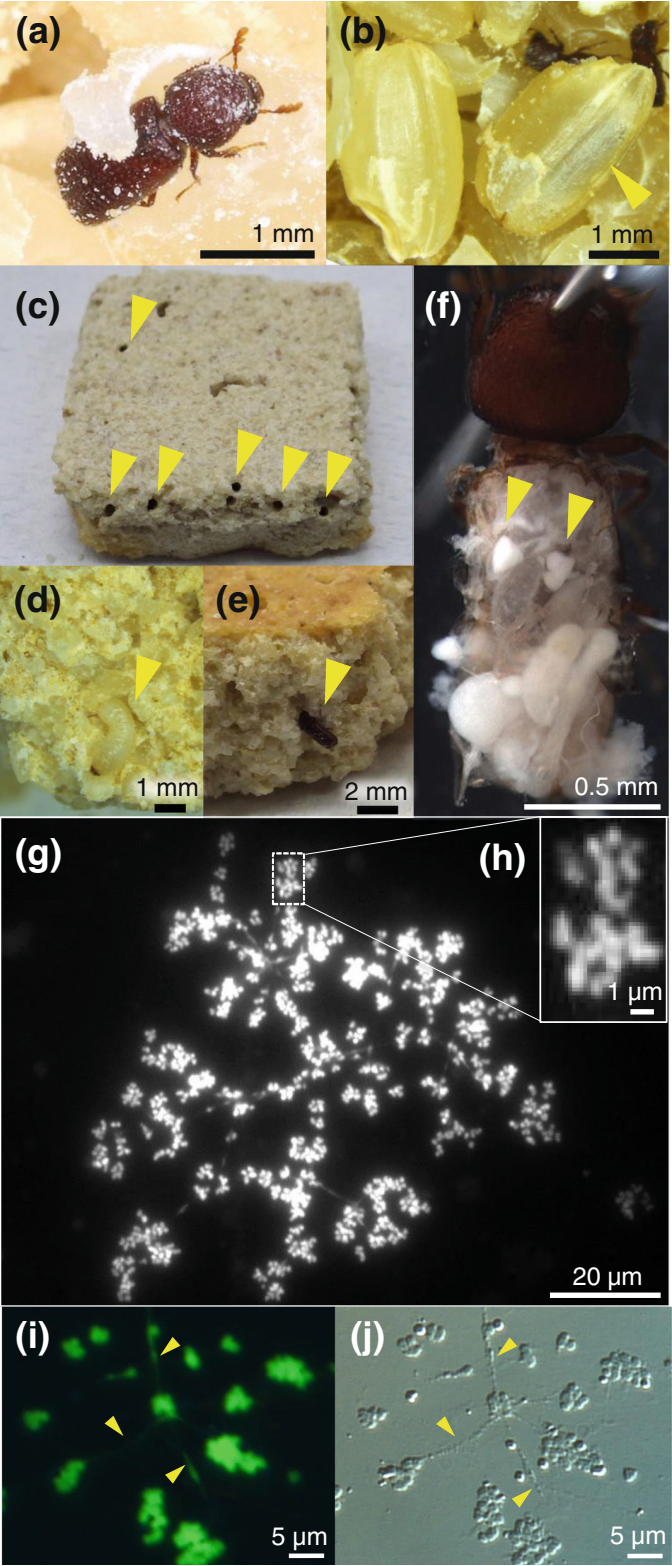
Morphology, bacteriomes and symbiotic bacteria of *R. dominica*. **a** An adult feeding on a rice grain. **b** A teneral adult inside a rice grain (arrowhead). **c** An artificial nutriment piece, approximately 3 cm × 3 cm × 1 cm in size, with holes tunneled by the insects (arrowheads). **d** A larva (arrowhead) in a broken diet piece. **e** A teneral adult (arrowhead) in a broken nutriment piece. **f** A dissected adult insect, in which the gut-associated paired bacteriomes are highlighted (arrowheads). **g-j** Microscopic images of symbiotic bacteria released from dissected bacteriomes and stained with SYTOX Green. **g** An epifluorescence microscopic image, in which rosette-like symbiotic bacteria can be seen. **h** A close-up of rosettes in **g**. **i** An enlarged epifluorescence microscopic image, highlighting the thread-like structures connecting the symbiotic bacteria (arrowheads). **j** A differential interference microscopic image corresponding to **i**

The lesser grain borer *Rhyzopertha dominica* (Coleoptera: Bostrichidae) (Fig. 1a), known as a cosmopolitan pest of stored grain, feeds on and breeds in rice, corn, wheat, and other starch-containing substrates [37]. The presence of a pair of oval bacteriomes in *R. dominica*, which are associated with the midgut and densely populated by pleomorphic bacterial cells of peculiar rosette-like shape, was first described by Mansour [34]. Later detailed histological descriptions were given by Buchner [35] and some experimental studies were reported by Huger [36]. Since this early work, however, no substantial studies have been conducted on this endosymbiotic system, except for several erroneous reports that claimed successful isolation, cultivation and analysis of the actually uncultivable bacterial symbiont [38–41].

In the present study, we investigated the microbiological nature, fine structure, intra-host localization, and infection dynamics of the peculiar bacterial symbiont of *R. dominica* in detail, using modern molecular, histological and microscopic techniques.

## Methods

### Insect and rearing

A long-lasting laboratory strain of *R. dominica* RdNFRI, which is of unknown origin and has been maintained on unpolished rice grains for over 20 years, was reared at 25 °C under constant darkness and used in this study. Collection of undamaged larvae, pupae and teneral adults of *R. dominica* from infested rice grains is difficult (Fig. 1b), so we developed an artificial diet rearing system for that purpose. Using an electric coffee mill, 90 g of unpolished rice grains were ground into a coarse powder, which we combined with 10 g of whole wheat flour and kneaded with 100 ml of water. The resultant dough was poured into about 3 cm × 3 cm square box-shaped molds made of aluminum foil, the poured molds were dried in an heating incubator at 65 °C for two days, and biscuit-like artificial diet pieces were obtained (Fig. 1c). Adult insects fed on, dug into, oviposited on and reproduced in the artificial nutriment, and we were readily able to obtain larvae, pupae and teneral adults by breaking apart the substrate (Fig. 1d, e). In the present study, 10% whole wheat flour was used as a binding agent as well as a food substrate. The insects readily accepted nutriment pieces containing either 0% or 50% whole wheat flour. However, the 0% wheat pieces were so fragile that the insect’s feeding activity resulted in their disintegration, whereas the 50% wheat pieces were too hard to be broken by hand for the purpose of obtaining larvae and pupae. Sexing of adult insects was conducted by a squeezing method as described previously [42, 43]. The abdomen of each adult insect was gently pressed and squeezed with forceps from anterior to posterior, exposing the genitalia from the abdominal tip. Squeezing was unnecessary to discern some, if not all, adult females, as the tips of their genitalia are always slightly exposed.

### DNA analysis

Adult insects were individually dissected in 70% ethanol, and the dissected bacteriomes were subjected to DNA extraction using QIAamp DNA Mini Kit (Qiagen). A 1.5 kb region of bacterial 16S rRNA gene was amplified from the DNA samples by PCR using the primers 10FF (5′-AGT TTG ATC ATG GCT CAG GAT-3′) and 1515R (5′-GTA CGG CTA CCT TGT TAC GAC TTA G-3′) [44] under the temperature profile of 94 °C for 5 min followed by 35 cycles of 94 °C for 30 s, 55 °C for 30 s and 72 °C for 1 min and a final incubation at 72 °C for 5 min. A smaller 0.6 kb region of bacterial 16S rRNA gene was also amplified by PCR using the primers 16SA2 (5′-GTG CCA GCA GCC GCG GTA ATA C-3′) and 16SB2 (5′-CGA GCT GAC GAC ARC CAT GCA-3′) [45] under the same temperature profile. The PCR products were electrophoresed in agarose gels and stained with ethidium bromide, and the amplified bands were excised from the gels and subjected to DNA extraction using the QIAquick Gel Extraction Kit (Qiagen). The purified PCR products were cloned using pT7Blue T-vector (Novagen) and *Escherichia coli* competent cells, and subjected to DNA sequencing using BigDye Terminator v3.1 Cycle Sequencing Kit (Applied Biosystems) and 3130xl Genetic Analyzer (Applied Biosystems).

### Molecular phylogenetic and evolutionary analyses

Nucleotide sequences were multiple-aligned using Clustal W [46] implemented in MEGA v7.0.26 [47]. The alignment was then inspected and corrected manually. Molecular phylogenetic analyses were conducted by neighbor-joining, maximum-likelihood, and Bayesian methods. Neighbor-joining phylogenies were constructed using MEGA v7.0.26 [47] with 1000 bootstrap replicates. The best-fit substitution model for the aligned sequences was evaluated by Kakusan v4 [48], which selected the GTR Gamma model for both the maximum-likelihood and Bayesian methods. Maximum-likelihood phylogenies were constructed using MEGA v7.0.26 [47] with 1000 bootstrap replicates. Bayesian phylogenies were inferred using MrBayes v3.2.6 [49]. Relative rate tests were performed by RRTree [50].

### Histological procedures

For observing fresh cell images of the symbiont, the bacteriomes were dissected from teneral adult insects using fine forceps in PBS [0.8% NaCl, 0.02% KCl, 0.115% Na_2_HPO_4_ and 0.02% KH_2_PO_4_ (*w/v*)], placed on a glass slide with a drop of SYTOX Green (Thermo Fisher Scientific) solution (1/1000 dilution), smashed with a coverslip, and observed under an epifluorescence microscope (Axiophot, Zeiss). For whole-mount fluorescence in situ hybridization, the insect samples were processed in 70% ethanol under a dissection microscope in order to facilitate infiltration of hybridization reagents. For adult insects, all wings were removed by forceps, and a side of the abdomen was cut with a razor. As for larvae and pupae, several holes were made by a needle at the anterior and posterior regions of the body. Eggs were treated with 50% bleach for 2 min followed by thorough washing with distilled water. These pre-treated insect samples were fixed in Carnoy’s solution (60% ethanol, 30% chloroform and 10% acetic acid) for at least 1 h at room temperature, washed thoroughly with 70% ethanol, and stored in 70% ethanol at 4 °C until use.

### Fluorescence in situ hybridization

Fluorescence in situ hybridization specifically targeting 16S rRNA of the symbiont was conducted essentially as described [51]. Briefly, the samples were rehydrated with PBT [PBS containing 0.1% Tween 20 (*v*/*v*)], and hybridized with hybridization buffer [20 mM Tris-HCl (pH 8.0), 0.9 M NaCl, 0.01% sodium dodecyl sulfate and 30% formamide (*w*/*v*)] containing 100 pmol/ml probe (5′-AlexaFluor555-TAT AGT TAC CTA CTC GCA AC-3′) at room temperature overnight. After washing with PBT three times for 10 min each at room temperature, the samples were placed on glass slides, mounted in 90% glycerol, and observed under a fluorescence dissection microscope (M165FC, Leica) and/or a laser scanning confocal microscope (LSM710, Zeiss). Digital images were merged and adjusted manually using Gimp Ver. 2.8 (GNU project).

### Electron microscopy

Teneral adult insects were dissected in 2.5% glutaraldehyde in 0.1 M phosphate buffer (pH 7.4), and the dissected bacteriomes were pre-fixed with the fixative at 4 °C overnight. Subsequently, the samples were post-fixed with 2% osmium tetroxide in 0.1 M phosphate buffer (pH 7.4) at 4 °C for 60 min, dehydrated through a water-ethanol series, embedded in Epon812 resin, processed into ultrathin sections (around 80 nm thick) by an ultramicrotome (EM UC7, Leica), mounted on copper meshes, stained with uranyl acetate and lead citrate, and observed under a transmission electron microscope (H-7600, Hitachi).

## Results and discussion

### Rosette-shaped symbiont in paired bacteriomes

In dissected teneral adult insects, paired oval bacteriomes were found in association with an anterior region of the midgut (Fig. 1f). When the dissected bacteriomes were crushed on a glass slide and observed under an epifluorescence microscope with DNA-staining fluorochrome, numerous symbiont cells of peculiar shape, flower- or rosette-like in appearance with radially connected lobes, were observed as clusters (Fig. 1g, h). It was difficult to determine whether each lobe represented a bacterial cell, each rosette constitutes a bacterial cell, or multiple rosettes are connected by thin DNA-positive filaments (presumable cytoplasmic bridges across rosettes; arrowheads in Fig. 1i, j) forming an extremely extended bacterial cell. The very peculiar morphology of these symbionts is thus consistent with early histological descriptions of the endosymbiotic system of *R. dominica* and other bostrichid species [34–36].

### Bacterial 16S rRNA gene of the symbiont

From the dissected bacteriomes, DNA was extracted and subjected to PCR, cloning and sequencing of bacterial 16S rRNA gene, which yielded identical 1578 bp sequences from three insects (sequence accession number LC310894). BLASTN searches against the DNA databases using the sequence as query retrieved 16S rRNA gene sequences of *Sulcia mulleri*, the ancient endosymbiont clade associated with diverse hemipteran insects (ex. sequence accession numbers AB772235, AB772237 and AB772238) [44, 52], as the top hits. In addition, a shorter segment of 16S rRNA gene was amplified by PCR and cloned from two insects. When 12 clones from each insect were sequenced, all 24 sequences, 583 bp in size, were identical. These results indicate that a single bacterial species dominates the endosymbiotic microbiota of *R. dominica.*

### Phylogenetic placement of the symbiont

**Fig. 2:**
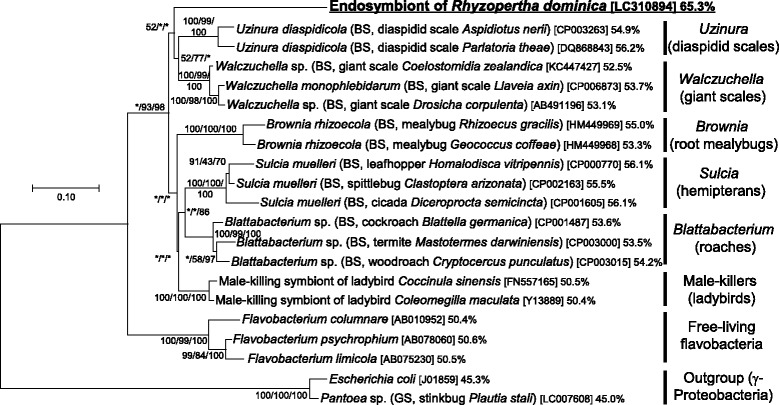
Phylogenetic placement of the symbiont of *R. dominica* in the Bacteroidetes based on 16S rRNA gene sequences. A maximum-likelihood phylogeny inferred from 1185 aligned nucleotide sites is shown. Statistical support values for each clade are indicated in the order of bootstrap probability of the neighbor-joining analysis, bootstrap probability of the maximum-likelihood analysis, and posterior probability of the Bayesian analysis from left to right, in which asterisks indicate values less than 50%. Host-related information in parentheses, accession number in brackets, and AT-content in percentage follow each bacterial taxon. BS and GS indicate bacteriocyte symbiont and gut symbiont, respectively. Labels for each major clade are shown on the right

Molecular phylogenetic analysis of the 16S rRNA gene sequence placed the bacterial symbiont of *R. dominica* within the bacterial phylum Bacteroidetes, in which the symbiont constituted a distinct lineage allied to a variety of insect-associated endosymbiont clades including *Uzinura* of diaspidid scales, *Walczuchella* of giant scales, *Brownia* of root mealybugs, *Sulcia* of diverse hemipterans, *Blattabacterium* of roaches, etc. (Fig. 2). These results suggest that *R. dominica* is associated with a novel bacterial endosymbiont belonging to the Bacteroidetes.

### In vivo localization and morphology of the symbiont

**Fig. 3:**
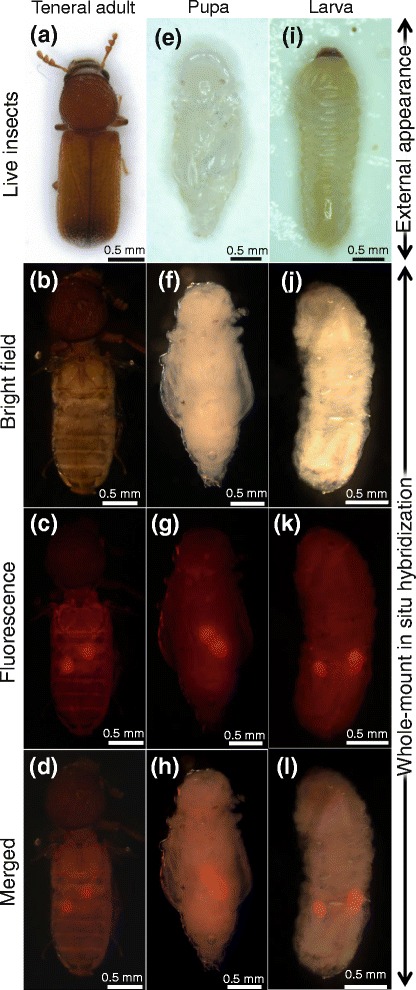
Localization of bacteriomes in adults, pupae and larvae of *R. dominica*. **a–d** Teneral adults. **e–h** Pupae. **i–l** Larvae. **a, e, i** Live insect images. **b, f, j** Bright field images of fixed and dissected insects subjected to whole-mount in situ hybridization specifically targeting the symbiont 16S rRNA. **c, g, k** Epifluorescence dissection microscopic images of the same insect samples as b, f and j, in which a pair of bacteriomes in the abdomen are visualized in red. **d, h, l** Merged images

**Fig. 4:**
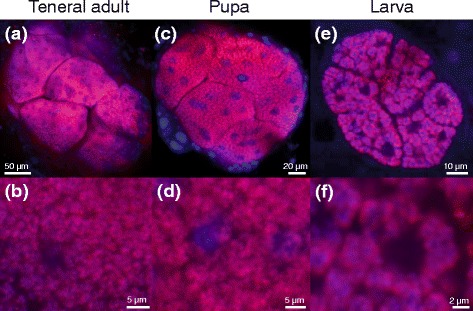
Laser confocal fluorescence microscopic images of bacteriomes and symbiotic bacteria of *R. dominica*. **a, b** Teneral adult. **c, d** Pupa. **e, f** Larva. **a, c, e** Images of dissected bacteriomes. **b, d, f** Enlarged images of bacteriocytes whose cytoplasm is full of symbiotic bacteria. Symbiont 16S rRNA is visualized by fluorescence in situ hybridization in red. DNA is counterstained in blue

**Fig. 5:**
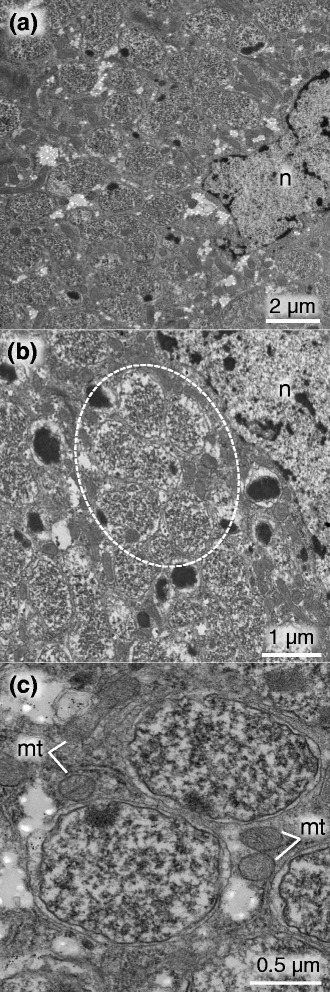
Transmission electron microscopic images of symbiotic bacteria of *R. dominica*. **a** A low magnification image of symbiotic bacteria in a bacteriocyte. **b** A rosette-like cluster of symbiont cells highlighted by a dotted circle. **c** A magnified image of symbiont cells. Abbreviations: n, nucleus; mt, mitochondrion

In order to visualize in vivo localization of the symbiont in *R. dominica*, we collected teneral adults (Fig. 3a), pupae (Fig. 3e) and larvae (Fig. 3i) from artificial nutriment (see Fig. 1d, e) and subjected them to whole-mount florescence in situ hybridization specifically targeting the symbiont 16S rRNA. In all teneral adults, pupae, and larvae, a pair of bacteriomes was consistently detected in the abdomen (Fig. 3). Confocal imaging of the dissected bacteriomes visualized dense population of the symbiotic bacteria, which were amorphous in shape presumably reflecting the rosette-like structure, in the cytoplasm of the bacteriocytes (Fig. 4). Transmission electron microscopy of the bacteriomes revealed the fine structure of the symbiotic bacteria (Fig. 5). In the cytoplasm of the bacteriocyte, the bacterial cells were enclosed by double membranes, where the outer membrane probably represents the host-derived symbiosomal membrane (Fig. 5c). The bacterial cells formed rosette-like clusters (Fig. 5a, b), in co-occurrence with numerous mitochondria (Fig. 5c).

### Infection dynamics of the symbiont

**Fig. 6:**
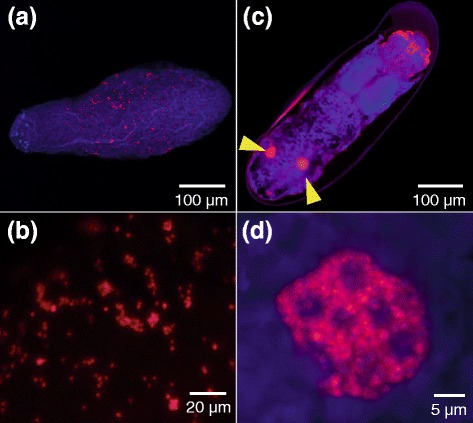
Localization of symbiotic bacteria in egg and embryo of *R. dominica*. **a** An egg, in which scattered symbiont signals are visible. **b** An enlarged image of the symbiont signals. **c** A late embryo, in which a pair of bacteriomes is already formed in the abdomen. **d** An enlarged image of the embryonic bacteriome. Symbiont 16S rRNA is visualized by fluorescence in situ hybridization in red, while DNA is counterstained in blue

**Fig. 7:**
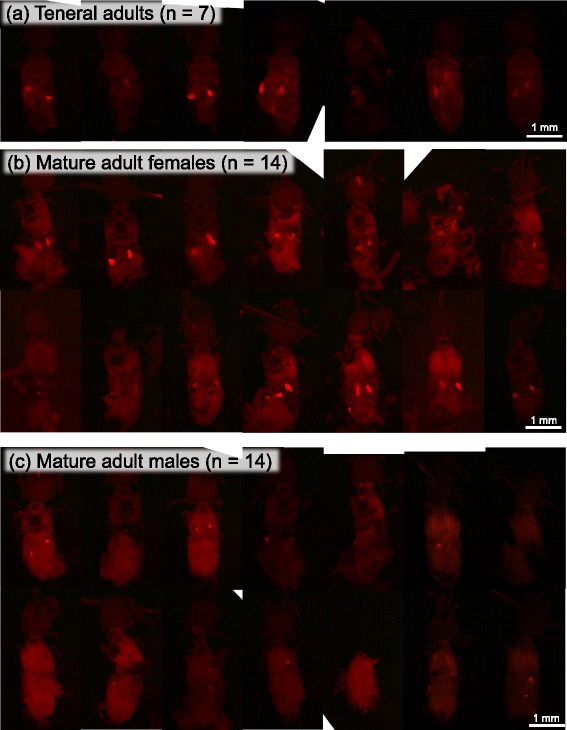
Whole-mount fluorescence in situ hybridization of adult insects of *R. dominica* for visualizing size and location of the bacteriomes. **a** Teneral adults (*n* = 7). **b** Mature adult females (*n* = 14). **c** Mature adult males (*n* = 14). Symbiont 16S rRNA is stained in red, highlighting the structure of the bacteriomes. All images are taken under an epifluorescence dissection microscope. Some images lack corners where the samples were located near the edge of microscopic visual fields

Fluorescence in situ hybridization identified scattered symbiotic bacteria within eggs (Fig. 6a, b) and formation of paired bacteriomes in embryos (Fig. 6c, d), which confirmed continuous host-symbiont association and vertical symbiont transmission in *R. dominica* as reported in previous studies [34–36]. The early histological observations noted that, specifically in adult males, the bacteriomes and the symbiotic bacteria therein tend to be atrophied and degenerated [35, 36]. Our whole-mount in situ hybridization data generally agreed with the previous reports, in that most adult males exhibited degeneration of the bacteriomes, although we found that some adult females also showed degeneration of the bacteriomes to some extent (Fig. 7). Notably, similar male-specific symbiont degeneration has been reported from diverse insects, including aphids [53–57], mealybugs [58], stictococcid scales [59], bird lice [60], weevils [61], etc., which may be relevant to vertical symbiont transmission in the host matrilines and/or sex-related differences in the host’s physiological dependency on the symbiont. Considering that only adult females are involved in vertical symbiont transmission to the offspring, it may be unnecessary for adult males to maintain this otherwise costly endosymbiotic system. Since adult females require much more energy and resources for producing eggs than adult males do for producing sperm, symbiont-provisioned nutrients such as amino acids and vitamins might be essential for adult females but not for adult males.

### Molecular evolutionary aspects of the symbiont

On the phylogenetic tree of the 16S rRNA gene sequence, the branch of the symbiont of *R. dominica* was remarkably elongated in comparison with that of other endosymbiont lineages in the Bacteroidetes, such as *Uzinura*, *Walczuchella*, *Brownia*, *Sulcia* and *Blattabacterium* (Fig. 2). The AT-content of the 16S rRNA gene sequence of the symbiont of *R. dominica*, 65.3%, was strikingly higher than those of the other endosymbiont lineages in the Bacteroidetes such as 55–56% for *Uzinura*, 52-53% for *Walczuchella*, 53-55% for *Brownia*, 55-56% for *Sulcia* and 53–54% for *Blattabacterium* (Fig. 2). Relative rate test based on the 16S rRNA gene sequence confirmed significantly accelerated molecular evolution in the symbiont of *R. dominica* in comparison with the insect endosymbiont lineages *Sulcia* and *Blattabacterium* (Table 1). It should be noted that these allied endosymbiont lineages are of ancient evolutionary origin, co-speciating with their hosts, and significantly genome-reduced, namely 0.26 Mb for *Uzinura* [62], 0.31 Mb for *Walczuchella* [63], 0.19-0.29 Mb for *Sulcia* [52, 64, 65], and 0.59-0.64 Mb for *Blattabacterium* [66–68]. It is thus plausible to speculate that the symbiont of *R. dominica* would also exhibit a high level of genome degeneration and reduction.

**Table 1:** Relative rate tests of 16S rRNA gene sequences of the symbiont of *Rhyzopertha dominica* in comparison with *Sulcia* symbionts and *Blattabacterium* symbionts

Lineage 1	Lineage 2	Outgroup	K1^*a*^	K2 ^*b*^	K1-K2	K1/K2	*P* value ^*c*^
Symbiont of *Rhyzopertha dominica* [LC310894]	*Sulcia* symbionts ^*d*^	*Flavobacterium columnare* [AB010952]	0.150	0.076	0.074	2.0	2.5 × 10^−6^
Symbiont of *Rhyzopertha dominica* [LC310894]	*Blattabacterium* symbionts ^*e*^	*Flavobacterium columnare* [AB010952]	0.159	0.059	0.100	2.7	1.0 × 10^−7^
*Sulcia* symbionts ^*d*^	*Blattabacterium* symbionts ^*e*^	*Flavobacterium columnare* [AB010952]	0.083	0.056	0.027	1.5	0.01

^a^Estimated mean distance between lineage 1 and the last common ancestor of lineages 1 and 2

^b^Estimated mean distance between lineage 2 and the last common ancestor of lineages 1 and 2

^c^ *P*-value was generated using the program RRTree [50]. The analyses were performed using 1445 aligned nucleotide sites for 16S rRNA gene sequences

^d^16S rRNA sequences of *Sulcia* symbionts from *Homalodisca vitripennis* (CP000770), *Diceroprocta semicincta* (CP001605) and *Clastoptera arizonana* (CP002163)

^e^16S rRNA sequences of *Blattabacterium* symbionts from *Blattella germanica* (CP001487), *Mastotermes darwiniensis* (CP003000), and *Cryptocercus punctulatus* (CP003015)

### Independent evolutionary origins of rosette-shaped insect symbionts

Apart from the rosette-shaped endosymbionts of the bostrichid beetles including *R. dominica* [34–36], an old histological study on endosymbionts of leaf beetles described a rosette-shaped bacterial symbiont associated with gut symbiotic organs and female genital accessory organs of *Bromius obscurus* (Coleoptera: Chrysomelidae) [69]. The similarity of the peculiar symbiont morphology, despite the phylogenetically distant insect hosts, raises the question of whether the rosette-shaped symbiotic bacteria of bostrichid beetles are phylogenetically related to those of the chrysomelid beetle [1]. Recently, molecular phylogenetic analyses showed that the symbiont of *B. obscurus* is a member of the class γ-Proteobacteria [70, 71]. We show in the present study that the symbiont of *R. dominica* belongs to the Bacteroidetes, which is phylogenetically distant from the γ-Proteobacteria (see Fig. 2). These results strongly suggest that the rosette-shaped pleomorphic symbionts have evolved independently from different bacterial groups in the Bostrichidae and the Chrysomelidae. Such amorphous bacterial morphology, known as the L-form, has been reported to occur when bacterial genes involved in cell wall synthesis and/or cell division are disrupted [72, 73]. Considering that both the rosette-shaped symbionts of *R. dominica* and *B. obscurus* exhibit remarkably AT-biased nucleotide compositions and significantly accelerated rates of molecular evolution [71] (see Fig. 2 and Table 1), the parallelism giving rise to the rosette-like bacterial shape may be attributable to degenerative evolution of the symbiont genomes.

## Conclusions

In this study, we demonstrated that the pleomorphic symbiont of *R. dominica*, whose microbiological affiliation has been obscure for decades, constitutes a distinct bacterial lineage in the Bacteroidetes. The Bacteroidetes embraces a variety of endosymbiont clades associated with plant-sucking insects (*Uzinura*, *Walczuchella* and *Brownia* of scale insects; *Sulcia* of diverse hemipterans) and omnivorous/xylophagous insects (*Blattabacterium* of cockroaches, woodroaches and termites) [74]. Here we add the endosymbiont of the grain-feeding insect, *R. dominica*, to the list of endosymbiont lineages that have evolved in the Bacteroidetes.

The peculiar rosette-like morphology of the symbiont of *R. dominica* is of particular interest. We found that the symbiont gene exhibits remarkably AT-biased nucleotide composition and significantly accelerated molecular evolution, which are suggestive of degenerative evolution of the symbiont genome. A morphologically similar bacterial symbiont was also reported from a chrysomelid leaf beetle [69], but that symbiont was recently shown to be a member of the γ-Proteobacteria [70, 71], suggesting that these rosette-shaped bacterial symbionts have independent evolutionary origins. The rosette-shaped symbiont of the leaf beetle also exhibited a remarkably AT-biased nucleotide composition and a significantly accelerated rate of molecular evolution [71]. We speculate that the reductive genome evolution, which is universally observed in bacteriocyte-associated endosymbionts of diverse insects [2, 64], may have resulted in disruption of bacterial genes involved in cell wall synthesis and/or cell division, which may be causative of the L-form-like amorphous morphology of the symbiont cells. Genome sequencing of the symbiont of *R. dominica* will provide information essential to addressing this intriguing evolutionary question. Biological roles of the symbiont for *R. dominica* are currently unknown and to be investigated in the future, for which the symbiont genome information, in combination with physiological data on experimentally generated symbiotic and aposymbiotic insects, will provide essential clues.

In addition to *R. dominica*, early histological studies described diverse bostrichid species, including *Apate degener*, *A. monachus*, *Bostrychoplites zickeli*, *Scobicia chevrieri*, *Sinoxylon ceratoniae* and *S. sexdentatus*, possessing similar paired bacteriomes and pleomorphic symbiotic bacteria therein [34, 35], raising the possibility that endosymbiotic association may have been inherited from a common ancestor. Surveys of these and other diverse bostrichid species for their endosymbiotic bacteria and co-phylogenetic analysis of the host-symbiont relationships would help to provide an integrated picture of the host–symbiont co-evolutionary history in the Bostrichidae.
